# Practical Formulation-Associated Immunomodulatory Responses of *Lacticaseibacillus paracasei* Yb in an Ovalbumin-Induced Allergic Airway Inflammation Mouse Model

**DOI:** 10.3390/microorganisms14071389

**Published:** 2026-06-23

**Authors:** Yi-Fang Ho, Tsung-Cheng Lee, Kai-Wei Liu, Fang-Yu Zhang, Chi-Yu Yang, Muhammet Ali Asan, Yu-Yi Chen, Yen-Po Chen, Tzu-Ying Chen

**Affiliations:** 1 Uni-President Enterprises Corporation, Tainan 710401, Taiwan; 2 Agricultural Input and Product Valorization Research Center, Agriculture Technology Research Institute, Miaoli 350401, Taiwan; 3 Department of Animal Science, National Chung Hsing University, Taichung City 40227, Taiwan; 4 The iEGG and Animal Biotechnology Research Center, National Chung Hsing University, Taichung City 40227, Taiwan

**Keywords:** *Lacticaseibacillus paracasei*, ovalbumin sensitization, fermented dairy product, freeze-dried yogurt, bacterial powder, immunomodulation

## Abstract

This study evaluated the immunomodulatory activity of live *Lacticaseibacillus paracasei* Yb in vitro and compared response patterns associated with practical *L. paracasei* Yb formulation formats in an ovalbumin (OVA)-induced allergic airway inflammation mouse model. In vitro, live *L. paracasei* Yb increased TNF-α production in RAW 264.7 macrophages at 2 × 10^6^ to 5 × 10^7^ CFU/mL, increased IL-1β only at 5 × 10^7^ CFU/mL, and increased IL-10 at 1 × 10^7^ and 5 × 10^7^ CFU/mL. In splenocytes, *L. paracasei* Yb increased TNF-α, IFN-γ, and IL-10 compared with untreated controls, although these responses did not show a simple concentration-dependent pattern. In vivo, BALB/c mice received fresh *L. paracasei* Yb yogurt (YG), freeze-dried yogurt (YG-FD), or bacterial powder (BP) for 53 days. Compared with the OVA-sensitized Negative control group, YG and BP did not significantly reduce serum total IgE or OVA-specific IgE, and airway responsiveness and BALF eosinophils showed limited or non-significant changes. In contrast, YG and BP significantly reduced lung inflammation scores (Negative control, 6.86 ± 1.57; YG, 5.13 ± 0.83; BP, 4.50 ± 0.55) and ConA-stimulated splenocyte IL-4 secretion (Negative control, 1168.43 ± 553.34 pg/mL; YG, 589.84 ± 233.54 pg/mL; BP, 472.28 ± 186.44 pg/mL). These findings suggest that practical formulation conditions may shape selected preclinical immunological and histopathological responses to *L. paracasei* Yb. Further studies incorporating CFU-matched dosing, probiotic-free yogurt controls, and mechanistic validation are required before clinical relevance in asthma can be inferred.

## 1. Introduction

Asthma is a chronic inflammatory respiratory disease and remains a major global health concern, with an estimated 358 million affected individuals worldwide [1]. The hygiene hypothesis has been proposed as one explanation for the rising prevalence of allergic diseases, suggesting that reduced early-life microbial exposure may contribute to immune dysregulation and allergic sensitization [2]. Although corticosteroids, leukotriene antagonists, and other pharmacological therapies are widely used to control asthma symptoms, concerns regarding long-term medication use have encouraged interest in adjunctive strategies that may help modulate allergic airway inflammation.

Probiotics, defined by the World Health Organization as “live microorganisms that, when administered in adequate amounts, confer a health benefit on the host,” have been studied for their role in regulating host immunity. Beyond effects on the gut microbiota and gastrointestinal function, probiotics can also influence intestinal and systemic immune responses [3]. Several studies suggest that selected probiotic strains may modulate allergic inflammation by altering the Th1/Th2 balance, dendritic cell activity, regulatory T cell responses, and microbiota-driven immune regulation, thereby reducing Th2-biased immune responses associated with allergic disease [4,5].

Among probiotic species, *Lacticaseibacillus paracasei* has attracted attention for its immunomodulatory properties [6,7]. Specific *L. paracasei* strains have been reported to improve allergic rhinitis symptoms [8,9] and reduce the risk or severity of atopic dermatitis [10]. These findings support the potential of *L. paracasei* as an immunomodulatory probiotic species. However, probiotic effects are strain-specific, and findings from one allergic condition or one strain should not be directly extended to another strain, formulation, or disease model without appropriate validation.

*Lacticaseibacillus paracasei* Yb is a probiotic strain with a history of use in food products and has received a health food permit in Taiwan for promoting beneficial gut bacteria and improving intestinal motility (Health Food Permit No. A00283, Ministry of Health and Welfare, Taiwan). A previous study by Lee et al. [11] suggested that *L. paracasei* Yb may modulate immune responses in ovalbumin (OVA)-sensitized mice, including reduced serum OVA-specific IgE levels and downregulation of Th2-associated cytokines. These findings support further preclinical evaluation of *L. paracasei* Yb in an OVA-induced allergic airway inflammation model, while its clinical efficacy in human asthma remains to be established.

The delivery form of probiotics is another important factor that may influence their biological effects. Many functional studies have evaluated probiotics as pure bacterial suspensions or powders, whereas practical applications commonly involve fermented dairy products, capsules, powders, or other food-based formulations. The formulation format may affect probiotic survival during gastrointestinal transit, reactivation after ingestion, metabolic activity, and host interactions [12]. Previous studies have shown that the overall formulation can modify probiotic efficacy and biological properties, but the direction and magnitude of this effect vary depending on the strain, product composition, processing method, and experimental model [13,14].

Despite this recognition, direct comparisons of practical probiotic formulation formats remain limited in models of allergic airway inflammation, creating a need to evaluate whether practical formulation choices influence the observed immunological effects of a given probiotic strain. The scientific gap extends beyond the presence of a food carrier alone. From a product-development perspective, probiotics are administered as complete formulations, such as fresh fermented yogurt, freeze-dried yogurt, or bacterial powder, each of which represents a distinct commercially feasible preparation pathway. These formulation pathways may influence probiotic viability, physiological state, post-ingestion reactivation, gastrointestinal exposure, and functional performance [12,15]. Previous studies have shown that probiotic efficacy can be affected by processing conditions, product environment, food components, and drying methods [12,16]. Therefore, different delivery formats should be considered not merely as vehicles for administration, but as total formulations that may shape downstream immunological readouts.

Based on this translational gap, the present study was designed to evaluate the immunomodulatory potential of *L. paracasei* Yb and to compare in vivo response patterns associated with practical formulation conditions under feasible dosing and gavage-volume constraints. First, we characterized the immunological responsiveness of RAW 264.7 macrophages and mouse splenocytes to live *L. paracasei* Yb at defined bacterial concentrations in vitro. Second, we compared in vivo immunological and tissue inflammatory responses after oral administration of three practical *L. paracasei* Yb-containing formulations, namely fresh yogurt, freeze-dried yogurt, and bacterial powder, in an OVA-induced allergic airway inflammation mouse model. This design was intended to distinguish *L. paracasei* Yb immune responsiveness observed in vitro from formulation-associated response patterns observed in vivo, rather than to isolate the yogurt matrix as a single experimental variable or to perform a CFU-matched mechanistic comparison.

## 2. Materials and Methods

### 2.1. Preparation of Probiotic Culture

*Lacticaseibacillus paracasei* Yb (BCRC 910415), originally isolated from infant feces, was preserved in a freeze-dried form until use. The strain was activated by inoculation into Lactobacilli MRS broth (Difco Laboratories, Detroit, MI, USA) and incubated aerobically at 37 °C for 24 h in an incubator (ASTEC, Fukuoka, Japan).

After incubation, the bacterial culture was centrifuged at 3000× *g* for 10 min at 4 °C, and the supernatant was discarded. The bacterial pellet was washed twice with sterile phosphate-buffered saline (PBS; Amresco, Solon, OH, USA) using the same centrifugation conditions. After washing, the bacterial cells were resuspended in sterile PBS. Viable bacterial suspensions were adjusted to 2 × 10^6^, 1 × 10^7^, and 5 × 10^7^ CFU/mL before being added to the cell culture assays. These concentrations served as live bacterial preparations for direct co-culture with immune cells.

### 2.2. Preparation for Mouse Feeding

*L*. *paracasei* Yb and the commercial lactic acid bacterial starter YF-L812 (Yo-Flex^®^ starter culture FD-DVS YF-L812, Chr. Hansen, Hørsholm, Denmark) were inoculated into pasteurized reconstituted skim milk and incubated at 38 °C for 9 h. After fermentation, the *L. paracasei* Yb yogurt was stored at 4 °C until use for oral administration.

For the freeze-dried yogurt formulation (YG-FD), the yogurt was freeze-dried and then ground into powder. Lyophilization was performed using a FreeZone Console Freeze Dryer with Stoppering Tray Dryer (Labconco, Kansas City, MO, USA) under vacuum (0 mbar) with a stepwise shelf-temperature program: −40 °C for 4 h, −30 °C for 12 h, −25 °C for 10 h, −20 °C for 12 h, −15 °C for 10 h, −10 °C for 12 h, −5 °C for 10 h, 0 °C for 6 h, followed by holding at 4 °C until collection. The recovery yield from the freeze-drying process was 13%. Given that the maximum reconstitution concentration of the freeze-dried yogurt was 410 mg/mL, the highest feasible daily dose within the limits of gavage volume was 164 mg/mouse per day, reconstituted in 0.4 mL of sterile water. The yogurt formulation (YG) was administered in its original form. The bacterial powder formulation (BP), comprising *L. paracasei* Yb powder supplied by Glac Biotech (Tainan, Taiwan), was suspended in sterile water immediately prior to administration. All prepared samples were maintained on ice and utilized on the day of preparation.

In accordance with animal welfare considerations regarding oral gavage, the administration volume was restricted to 10 mL/kg per gavage. For a 20 g mouse, this equates to a maximum of 0.2 mL per administration. The mice were subjected to gavage twice daily, resulting in a total daily dosage of 0.4 mL per animal. The formulations tested and the corresponding daily doses are detailed in Table 1. Body-weight-normalized doses were calculated assuming a 20 g mouse.

Viable *L. paracasei* Yb counts in the administered preparations were quantified using MRS agar. Plates were incubated at 37 °C under anaerobic conditions for 72 h.

### 2.3. Cell Culture and Co-Culture Model

The in vitro assays were designed to characterize the immune responsiveness to live *L. paracasei* Yb at defined bacterial concentrations.

RAW 264.7 cell line

RAW 264.7 mouse macrophages were obtained from the Bioresource Collection and Research Center (BCRC, Hsinchu, Taiwan). Cells were cultured in Dulbecco’s Modified Eagle’s Medium (DMEM; Gibco, Grand Island, NY, USA) supplemented with 10% heat-inactivated fetal bovine serum (FBS; 56 °C for 30 min, Sigma-Aldrich, St. Louis, MO, USA) and 1% Antibiotic-Antimycotic solution (10,000 U/mL penicillin, 10,000 μg/mL streptomycin, and 25 μg/mL amphotericin B; Gibco). Cells were maintained at 37 °C in a humidified incubator containing 5% CO_2_.

Splenocytes

Splenocytes were isolated from 6-week-old female C57BL/6 mice (BioLASCO, Taipei, Taiwan). Briefly, spleens were aseptically removed and passed through 70 μm nylon cell strainers (BD Falcon, Franklin Lakes, NJ, USA) using the plunger end of sterile syringes. The cell suspension was washed with 10 mL sterile PBS (Sigma-Aldrich) and centrifuged at 400× *g* for 5 min at 4 °C. After removal of the supernatant, erythrocytes were lysed by adding 5 mL of 1× RBC lysis buffer (Thermo Fisher Scientific, Waltham, MA, USA) and incubating for 2 min at room temperature. The suspension was then centrifuged at 400× *g* for 5 min, and the resulting pellet was resuspended in RPMI 1640 medium (Thermo Fisher Scientific) supplemented with 10% FBS.

For co-culture experiments, RAW 264.7 cells or splenocytes were seeded at a density of 1 × 10^5^ cells/well in 24-well plates and allowed to stabilize for 16 h before treatment. Live *L. paracasei* Yb cells were added directly to the cultures at a multiplicity of infection (MOI, bacteria:cell ratio) of 20:1 (2 × 10^6^ CFU/well), 100:1 (1 × 10^7^ CFU/well), and 500:1 (5 × 10^7^ CFU/well). Cells cultured without bacterial treatment served as the Control. After 24 h of co-culture, culture supernatants were collected and analyzed for TNF-α, IL-1β, IFN-γ, and IL-10 production using mouse cytokine ELISA kits (R&D Systems, Minneapolis, MN, USA) according to the manufacturer’s instructions.

### 2.4. Animals

Female, 6-week-old BALB/c specific pathogen-free mice were obtained from the National Laboratory Animal Center (Taiwan). A total of 40 mice, with body weights ranging from 18 to 25 g at the initiation of the study, were employed. The mice underwent a minimum one-week acclimation period prior to treatment and were housed in individually ventilated cages, with four mice per cage, in accordance with professional animal care standards.

Animals were provided with ad libitum access to rodent chow and water. The animal facility was maintained under a 12 h light/dark cycle at a temperature of 24 ± 2 °C and relative humidity of 50 ± 20%. Body weight and food intake were measured weekly. Prior to the commencement of treatment, mice were randomly allocated to experimental groups, and there were no significant differences in mean body weight among the groups (*p* > 0.05). Investigators were not blinded during animal handling and oral administration; however, histopathological evaluation was performed by an experienced pathologist blinded to treatment allocation.

All animal procedures were reviewed and approved by the Institutional Animal Care and Use Committee of the Agricultural Technology Research Institute (IACUC No. 111048). Animals were monitored throughout the study in accordance with the approved animal protocol. The study design, animal welfare considerations, and humane endpoints of the in vivo procedures were prepared with reference to the ARRIVE (Animal Research: Reporting of In Vivo Experiments) guidelines 2.0. No animals reached the predefined humane endpoints before scheduled sacrifice.

### 2.5. OVA-Induced Allergic Airway Inflammation Mouse Model

An OVA-induced allergic airway inflammation mouse model was used to evaluate the immunomodulatory and tissue inflammatory responses associated with oral administration of *L. paracasei* Yb formulations [17,18]. The mice were randomly allocated to five groups, with eight mice per group.

1. Control, healthy mice without OVA sensitization;
2. Negative control, OVA-sensitized mice receiving sterile water;
3. YG-FD, OVA-sensitized mice receiving freeze-dried *L. paracasei* Yb yogurt;
4. YG, OVA-sensitized mice receiving *L. paracasei* Yb yogurt;
5. BP, OVA-sensitized mice receiving *L. paracasei* Yb bacterial powder.

Test substances or sterile water were administered orally by gavage twice daily for 53 consecutive days, starting at 6 weeks of age. Each administration volume was 0.2 mL per mouse, for a total daily volume of 0.4 mL. Control and Negative control mice received sterile water. The YG-FD group received reconstituted freeze-dried yogurt, the YG group received yogurt, and the BP group received bacterial powder suspended in sterile water, as described in Section 2.2. The test substances were freshly prepared on the day of administration, kept on ice after preparation, and administered via gavage.

For systemic sensitization, all mice, except the Control group, were sensitized by intraperitoneal injection of OVA adsorbed to alum adjuvant (Imject™ alum adjuvant, Sigma). Each mouse was administered 0.2 mL of OVA containing 2 μg per injection. The initial sensitization occurred on day 29, with a subsequent session on day 43. Control mice received PBS in place of OVA during the sensitization procedure. This systemic OVA/alum sensitization step was adapted from established OVA-induced allergic airway inflammation models [17,18].

On days 50–52, mice in the OVA-sensitized groups were challenged intratracheally with 50 μL of 3% OVA in sterile saline, whereas mice in the Control group received 50 μL of sterile saline. Prior to intratracheal delivery, mice were anesthetized with 4-5% isoflurane in an acrylic chamber. The incisors of the mice were fixed to a vertical board, the tongue was carefully extended, and the tracheal opening was visualized using a laryngoscope. Subsequently, OVA or saline was administered intratracheally employing a microsprayer. This intratracheal OVA challenge procedure was adapted from a published mouse model of OVA-induced allergic airway inflammation [17].

**Figure 1:**
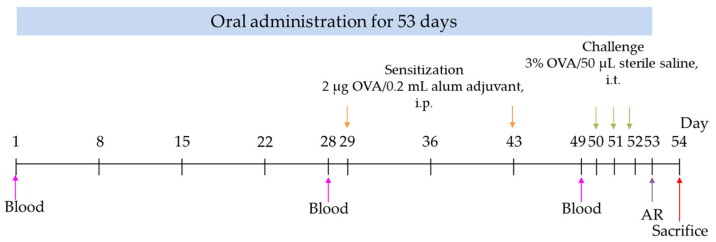
Experimental flowchart. OVA, ovalbumin; i.p., intraperitoneal; i.t., intratracheal; AR, airway responsiveness.

Blood samples were collected from the cheek vein on day 1 (prior to the animal experiment), before allergen induction (day 28), and on day 49 to measure OVA-specific IgE levels. Following the final OVA or saline challenge, airway responsiveness was monitored at 1 and 6 h post-challenge, with baseline measurements obtained prior to the challenge. The mice were euthanized 24 h after the final challenge to collect bronchoalveolar lavage fluid, lavaged lung tissue, spleen, and blood samples (Figure 1).

### 2.6. Airway Responsiveness

Airway responsiveness was assessed utilizing barometric unrestrained whole-body plethysmography (Buxco; EMKA Technologies, Paris, France) with a Vivo Flow whole-body, head-out, double-chamber system [19]. The apparatus was calibrated prior to each formal recording in accordance with the manufacturer’s guidelines, and measurement conditions were consistently maintained throughout all subjects. To minimize measurement variability, mice underwent three acclimation and pre-test sessions during the week preceding sacrifice, followed by a single formal measurement. During plethysmography procedures, the mice remained conscious and unrestrained, with no anesthesia administered throughout the recordings. Baseline airway responsiveness was recorded immediately before intratracheal administration (0 h). The control group received 50 μL of sterile normal saline per mouse, whereas the OVA-sensitized groups received 50 μL of 3% OVA per mouse. Airway responsiveness was subsequently evaluated at 1 and 6 h post-administration. Each mouse was observed for 15 min at each time point, with the initial 5 min allocated to chamber acclimatization. The primary respiratory parameter analyzed was enhanced pause (Penh), which served as an indicator of airway responsiveness [19].

### 2.7. Analysis of Bronchoalveolar Lavage Fluid

The lungs were lavaged three times with 0.5 mL of sterile PBS per lavage (total instilled volume, 1.5 mL). From each mouse, at least 1.0 mL of bronchoalveolar lavage fluid (BALF) was recovered, representing a lavage recovery rate of no less than 66.7%. The recovered BALF was centrifuged at 425× *g* at 4 °C for 15 min. The resulting cell pellet was gently resuspended in sterile PBS. Residual erythrocytes were eliminated using an RBC lysis solution, and the remaining leukocyte suspension was employed for cellular analysis. The BALF collection and subsequent cell-recovery procedures were adapted from an established murine bronchoalveolar lavage method to evaluate inflammatory cell infiltration [20].

BALF cell populations were analyzed using a hematology analyzer (ProCyte Dx, IDEXX Laboratories, Inc., Westbrook, ME, USA). The analysis included total white blood cell (WBC) counts and differential cell counts, including monocytes (MONOs) and eosinophils (EOSs). After lavage, the lungs were fixed in 10% neutral buffered formalin for subsequent histological examination.

### 2.8. Serum Immunoglobulin Measurement

After blood collection, samples were allowed to clot at ambient temperature for approximately 30 min, then centrifuged at 1300× *g* for 15 min at 4 °C. The resulting serum supernatant was carefully collected and stored at −80 °C until subsequent analysis. Serum levels of total IgE and OVA-specific IgE were quantified utilizing commercial mouse ELISA kits (Invitrogen, Waltham, MA, USA) in accordance with the manufacturer’s instructions. Briefly, serum samples were diluted according to the guidelines provided within each immunoglobulin assay kit. The dilution factor varied with the specific immunoglobulin measured; therefore, the recommended dilution ratios, as specified in the respective assay protocols, were strictly followed for each target analyte to ensure all measurements remained within the optimal detection range. Standards and samples were added to the ELISA plates in duplicate. The absorbance of each well was measured using a microplate reader at the wavelength recommended by the kit manufacturer, and immunoglobulin concentrations were calculated based on the corresponding standard curves. Standard curves were generated following the instructions provided in each assay kit. All immunoglobulin quantifications were performed using a four-parameter logistic (4PL) regression model, as specified by the manufacturer.

All analyses were conducted within 3 months of sample storage to mitigate potential degradation and ensure data reliability.

### 2.9. Splenocyte Stimulation and Cytokine Secretion Assay

Mouse spleens were harvested and placed onto sterile 200-mesh filters. Using the blunt end of a sterile syringe, spleens were gently disrupted while being flushed with RPMI 1640 medium. The cell suspension was centrifuged at 300× *g* and 4 °C for 10 min, and the supernatant was discarded. Erythrocytes were lysed by resuspending the pellet in HBSS (Sigma-Aldrich, USA) for 3 to 5 min. The cell suspension was then centrifuged again under the same conditions, and the pellet was resuspended in RPMI 1640 medium supplemented with 10% FBS (Thermo Fisher Scientific, USA), 2 mM L-glutamine (Thermo Fisher Scientific), and 2 mM sodium pyruvate (Thermo Fisher Scientific). After cell counting, the splenocyte suspension was adjusted to 2 × 10^7^ viable cells/mL.

For the stimulation assay, 50 µL of splenocyte suspension was seeded into each well of a 24-well plate, resulting in 1 × 10^6^ cells per well. A total of 950 µL of ConA-containing medium or control medium without ConA (Sigma-Aldrich) was added to each well, resulting in a final culture volume of 1 mL and a final ConA concentration of 5 µg/mL. ConA was used as a polyclonal T-cell mitogen to evaluate cytokine secretion capacity following ex vivo stimulation [21]. Plates were incubated at 37 °C in a humidified 5% CO_2_ incubator for 24 or 72 h.

At each endpoint, the cell suspension from each well was collected and centrifuged at 300× *g* for 10 min at room temperature. Supernatants were transferred to 1.5 mL microcentrifuge tubes and stored at −80 °C until cytokine analysis. For the 24 h samples, 200 µL of supernatant was used to measure TNF-α and IL-6. For the 72 h samples, 500 µL of supernatant was used to measure IL-4. Cytokine concentrations were determined using commercial mouse cytokine ELISA kits (Invitrogen, USA) according to the manufacturer’s instructions.

### 2.10. Histological Evaluation

After bronchoalveolar lavage was completed as described in Section 2.7, the lavaged lungs were collected and fixed in 10% neutral buffered formalin. The fixed lung tissues were processed for paraffin embedding, sectioned, and stained with hematoxylin and eosin. Histopathological evaluation was performed under a light microscope (Leica DM2700M, Wetzlar, Germany) by an experienced pathologist who was blinded to the treatment allocation. For each animal, one lung tissue section was examined at 100× magnification, and lesion severity was manually scored by the blinded pathologist based on the overall histopathological findings in the section.

Lung lesions were assessed using a semi-quantitative severity grading scheme based on the criteria described by Shackelford et al. [22]: 0 = not present; 1 = minimal (<1%); 2 = slight (1–25%); 3 = moderate (26–50%); 4 = moderately severe/high (51–75%); and 5 = severe/high (76–100%). Two lesion categories were evaluated in the lung tissue: diffuse inflammatory cell infiltration and diffuse granulomatous inflammatory response. Each lesion category was scored from 0 to 5, and the lung inflammation score was calculated as the sum of these two lesion scores. Therefore, the total lung inflammation score ranged from 0 to 10. Quantification was performed by semi-quantitative manual scoring under light microscopy.

### 2.11. Statistical Analysis

Data are expressed as means ± standard deviation (SD). Normality was assessed using the Shapiro–Wilk test. Differences among multiple groups were analyzed by one-way analysis of variance (ANOVA), followed by Tukey’s honestly significant difference (HSD) post hoc test for multiple comparisons. Differences were considered statistically significant when *p* < 0.05. Statistical analyses were performed using IBM SPSS Statistics (27.0.1.0).

## 3. Results

### 3.1. Effect of L. paracasei Yb on Cytokine Production in Macrophages and Splenocytes

**Figure 2:**
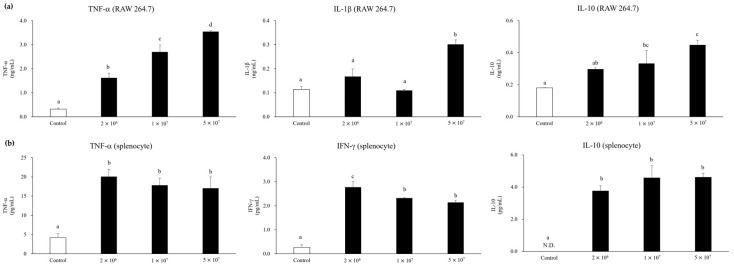
Effect of *Lacticaseibacillus paracasei* Yb on cytokine production in (**a**) murine RAW 264.7 macrophages and (**b**) murine splenocytes. ND, not detected. Data are expressed as means ± SD (n = 3). Different lowercase letters above the bars indicate significant differences among groups, as determined by one-way ANOVA followed by Tukey’s HSD post hoc test (*p* < 0.05).

To evaluate the in vitro immunomodulatory potential of *L. paracasei* Yb, RAW 264.7 macrophages and mouse splenocytes were incubated with live *L. paracasei* Yb for 24 h, and the concentrations of TNF-α, IL-1β, IFN-γ, and IL-10 were measured. In RAW 264.7 macrophages (Figure 2a), *L. paracasei* Yb increased TNF-α from 0.317 ± 0.043 ng/mL in the Control group to 1.621 ± 0.193, 2.695 ± 0.294, and 3.542 ± 0.037 ng/mL at 2 × 10^6^, 1 × 10^7^, and 5 × 10^7^ CFU/mL, respectively. IL-1β increased only at the highest concentration (0.302 ± 0.018 ng/mL) compared with the Control group (0.114 ± 0.012 ng/mL), whereas IL-10 increased from 0.180 ± 0.002 ng/mL in the Control group to 0.45 ± 0.03 ng/mL at 5 × 10^7^ CFU/mL, with intermediate values at 2 × 10^6^ and 1 × 10^7^ CFU/mL (0.297 ± 0.009 and 0.333 ± 0.081 ng/mL, respectively). In mouse splenocytes (Figure 2b), *L. paracasei* Yb also increased TNF-α, IFN-γ, and IL-10 compared with the Control group. TNF-α increased from 4.190 ± 1.029 pg/mL in the Control group to a range of 17.048 to 20.063 pg/mL across the three bacterial concentrations. IFN-γ increased from 0.268 ± 0.112 pg/mL to between 2.136 and 2.773 pg/mL. IL-10 was not detected in the Control group but was detectable after *L. paracasei* Yb stimulation, with values of 3.755 ± 0.325 pg/mL at 2 × 10^6^ CFU/mL and 4.569 ± 0.768 pg/mL at 1 × 10^7^ CFU/mL; the 5 × 10^7^ CFU/mL group was comparable to the 1 × 10^7^ CFU/mL group. Overall, these results indicate that live *L. paracasei* Yb activated cytokine responses in macrophages and splenocytes in vitro, although the splenocyte responses did not follow a simple concentration-dependent pattern.

### 3.2. Effect of Oral Administration of L. paracasei Yb on Serum IgE and OVA-Specific IgE in an OVA-Induced Allergic Airway Inflammation Model

**Figure 3:**
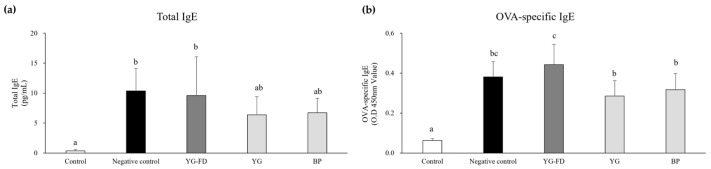
Effect of oral administration of *Lacticaseibacillus paracasei* Yb on (**a**) total IgE and (**b**) ovalbumin (OVA)-specific IgE production in OVA-sensitized BALB/c mice. YG-FD, freeze-dried *L. paracasei* Yb yogurt; YG, *L. paracasei* Yb yogurt; BP, *L. paracasei* Yb bacterial powder. Data are expressed as mean ± SD of 8 mice per group and were analyzed by one-way ANOVA followed by Tukey’s HSD post hoc test. Different lowercase letters indicate significant differences among groups (*p* < 0.05).

The effects of *L. paracasei* Yb formulations on serum immunoglobulin responses were assessed after 53 days of oral administration in the OVA-induced allergic airway inflammation model. As shown in Figure 3a, serum total IgE was lowest in the Control group (0.35 ± 0.24 pg/mL) and increased significantly in the Negative control group (10.35 ± 3.78 pg/mL). The YG-FD group showed a comparable total IgE level (9.60 ± 6.46 pg/mL), while the YG and BP groups showed lower average levels (6.36 ± 3.04 and 6.73 ± 2.40 pg/mL, respectively). However, YG and BP did not differ significantly from the Negative control group. For OVA-specific IgE (Figure 3b), the Control group had the lowest level (0.06 ± 0.01), whereas all OVA-sensitized groups showed elevated levels. Among these, YG and BP had lower OVA-specific IgE levels than YG-FD (0.29 ± 0.08 and 0.32 ± 0.08 vs. 0.44 ± 0.10), but none showed significant differences compared to the Negative control group (0.38 ± 0.08). These results show that YG and BP exhibited lower mean serum IgE and OVA-specific IgE levels than YG-FD, but these differences were not sufficient to demonstrate significant suppression relative to the Negative control group.

### 3.3. Airway Responsiveness in the OVA-Induced Allergic Airway Inflammation Model

**Figure 4:**
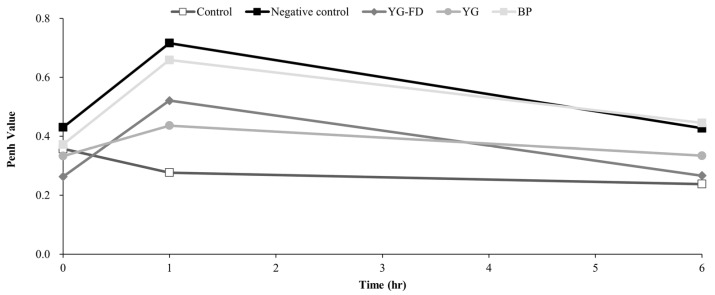
Effect of oral administration of *Lacticaseibacillus paracasei* Yb on airway responsiveness in BALB/c mice. Enhanced pause (Penh) was measured at 0, 1, and 6 h after intratracheal PBS or OVA challenge using whole-body plethysmography. YG-FD, freeze-dried *L. paracasei* Yb yogurt; YG, *L. paracasei* Yb yogurt; BP, *L. paracasei* Yb bacterial powder. Data are expressed as mean ± SD of 8 mice per group and were analyzed at each time point by one-way ANOVA followed by Tukey’s HSD post hoc test.

Airway responsiveness was evaluated by measuring enhanced pause (Penh) at 0, 1, and 6 h after the final intratracheal PBS or OVA challenge (Figure 4). At baseline (0 h), mean Penh values were generally comparable among groups, ranging from 0.26 ± 0.08 in the YG-FD group to 0.43 ± 0.21 in the Negative control group. At 1 h after challenge, the Negative control group showed the highest mean Penh value (0.72 ± 0.41), whereas the Control, YG-FD, YG, and BP groups showed mean values of 0.28 ± 0.04, 0.52 ± 0.51, 0.44 ± 0.32, and 0.66 ± 0.43, respectively. At 6 h after challenge, the Negative control group remained numerically higher than the Control group (0.43 ± 0.15 vs. 0.24 ± 0.04), while the YG-FD and YG groups showed lower mean Penh values (0.27 ± 0.06 and 0.33 ± 0.11, respectively) than the Negative control group. However, no significant pairwise differences were detected among groups at any time point. Thus, although YG-FD and YG showed numerically lower Penh values than the Negative control group after OVA challenge, these differences did not reach statistical significance.

### 3.4. Effect of L. paracasei Yb on Histological Changes

**Figure 5:**
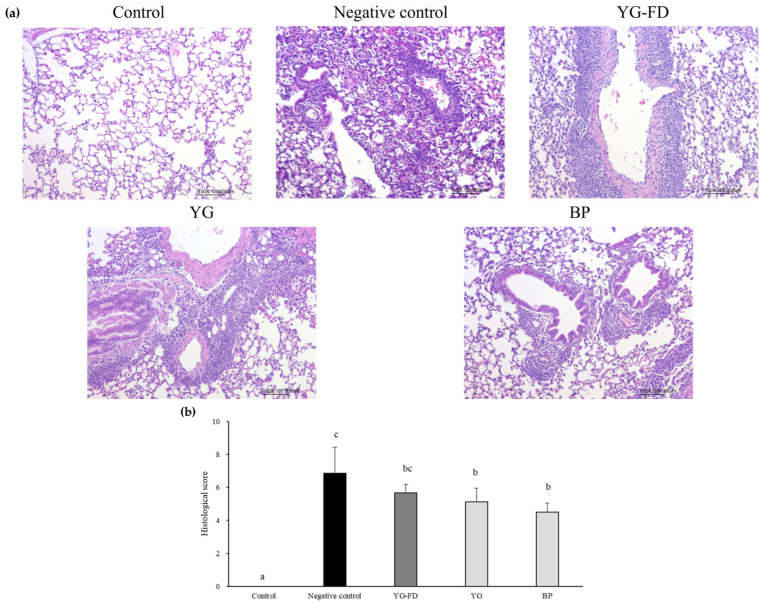
Effect of oral administration of *Lacticaseibacillus paracasei* Yb on (**a**) hematoxylin and eosin-stained mouse lung sections and (**b**) histological scores for lung tissues in BALB/c mice. YG-FD, freeze-dried *L. paracasei* Yb yogurt; YG, *L. paracasei* Yb yogurt; BP, *L. paracasei* Yb bacterial powder. Data are expressed as mean ± SD of 8 mice per group and analyzed by one-way ANOVA followed by Tukey’s HSD post hoc test. Different lowercase letters indicate significant differences among groups (*p* < 0.05).

Histological examination revealed no remarkable histopathological findings in the Control group (lung inflammation score, 0.00 ± 0.00). In contrast, the Negative control group exhibited pronounced lung inflammatory lesions, including inflammatory cell infiltration and granulomatous inflammatory responses (Figure 5a), with the highest mean lung inflammation score (6.86 ± 1.57; Figure 5b). Compared with the Negative control group, the YG and BP groups had significantly lower histological scores (5.13 ± 0.83 and 4.50 ± 0.55, respectively). The YG-FD group also had a lower mean score (5.67 ± 0.52) than the Negative control group, but this difference was not statistically significant. These results indicate that the YG and BP formulations were associated with reduced lung inflammatory lesions in the OVA-induced allergic airway inflammation model.

### 3.5. Effect of L. paracasei Yb on Cytokine Secretion in Splenocyte Cultures

**Figure 6:**
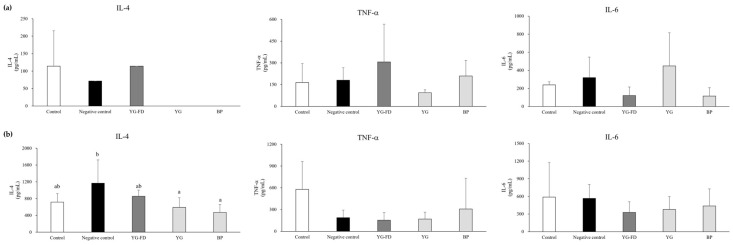
Effect of oral administration of *Lacticaseibacillus paracasei* Yb on cytokine production in splenocyte cultures from BALB/c mice. Cytokine levels were measured (**a**) without stimulation and (**b**) after ConA stimulation. YG-FD, freeze-dried *L. paracasei* Yb yogurt; YG, *L. paracasei* Yb yogurt; BP, *L. paracasei* Yb bacterial powder. Data are expressed as mean ± SD of 8 mice per group and were analyzed by one-way ANOVA followed by Tukey’s HSD post hoc test. Different lowercase letters indicate significant differences among groups (*p* < 0.05).

To assess systemic immune responses, splenocytes isolated from mice were cultured with or without ConA stimulation, and IL-4, TNF-α, and IL-6 levels in the culture supernatants were measured (Figure 6). Under unstimulated conditions, no significant differences were observed among groups for IL-4, TNF-α, or IL-6 (Figure 6a). TNF-α levels ranged from 94.14 ± 20.02 pg/mL in the YG group to 306.06 ± 260.37 pg/mL in the YG-FD group, while IL-6 levels ranged from 115.19 ± 92.47 pg/mL in the BP group to 449.20 ± 366.28 pg/mL in the YG group. Because these differences were not statistically significant, they were not interpreted as treatment-related changes. After ConA stimulation, the Negative control group showed the highest IL-4 level (1168.43 ± 553.34 pg/mL), whereas the YG and BP groups showed significantly lower IL-4 levels (589.84 ± 233.54 and 472.28 ± 186.44 pg/mL, respectively; Figure 6b). The Control and YG-FD groups showed intermediate IL-4 levels (715.47 ± 204.44 and 854.89 ± 147.17 pg/mL, respectively). In contrast, TNF-α and IL-6 levels after ConA stimulation did not differ significantly among groups. These results indicate that oral administration of YG and BP was associated with a lower ConA-stimulated IL-4 response, whereas TNF-α and IL-6 responses were not significantly altered.

### 3.6. Effect of L. paracasei Yb on Inflammatory Cells in BALF

**Figure 7:**
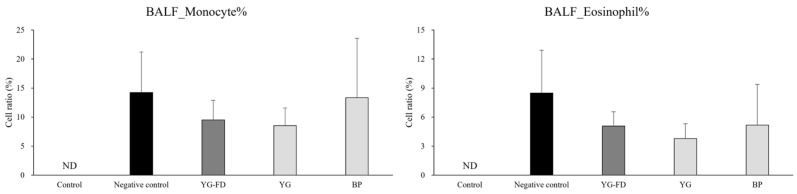
Effect of oral administration of *Lacticaseibacillus paracasei* Yb on the distribution of immune cells in bronchoalveolar lavage fluid (BALF) of BALB/c mice. YG-FD, freeze-dried *L. paracasei* Yb yogurt; YG, *L. paracasei* Yb yogurt; BP, *L. paracasei* Yb bacterial powder; ND, not detected. Data are expressed as mean ± SD of 8 mice per group and analyzed by one-way ANOVA followed by Tukey’s HSD post hoc test.

To evaluate inflammatory cell accumulation in the airways, monocyte and eosinophil proportions in BALF were measured (Figure 7). Because the Control group was not sensitized or challenged with OVA, monocytes and eosinophils were not detected in BALF and are indicated as ND (not detected). Among the OVA-sensitized groups, the mean monocyte percentages were 14.23 ± 6.99% in the Negative control group, 9.52 ± 3.38% in the YG-FD, 8.53 ± 3.03% in the YG, and 13.33 ± 10.22% in the BP group, with no significant differences observed. For eosinophils, the Negative control group had the highest mean percentage (8.47 ± 4.45%), while the YG group had the lowest (3.78 ± 1.54%) among the OVA groups. The YG-FD and BP groups showed intermediate values of 5.08 ± 1.48% and 5.18 ± 4.20%, respectively. No significant differences in eosinophil percentages were found among the OVA-sensitized groups. These findings suggest that *L. paracasei* Yb-containing formulations did not significantly affect BALF monocyte or eosinophil proportions under these experimental conditions, although the YG group displayed a numerically lower eosinophil percentage compared with the Negative control group.

## 4. Discussion

This study evaluated the immunomodulatory activity of *L. paracasei* Yb in vitro and examined whether its effects in a mouse model of OVA-induced allergic airway inflammation varied across three practical formulation formats: fresh yogurt (YG), freeze-dried yogurt (YG-FD), and bacterial powder (BP). The in vitro experiments showed that live *L. paracasei* Yb activated RAW 264.7 macrophages and splenocytes, as reflected by changes in TNF-α, IL-1β, IFN-γ, and IL-10 production. In the animal model, the treatment effects were endpoint-specific rather than uniformly significant across all allergic airway inflammation-related outcomes. YG and BP significantly reduced lung histological scores and ConA-stimulated splenocyte IL-4 secretion, whereas serum total IgE, OVA-specific IgE, airway responsiveness, and BALF eosinophils showed numerical or intermediate changes that did not consistently reach statistical significance. Therefore, the current findings support a cautious interpretation that *L. paracasei* Yb may modulate selected immune and tissue inflammatory responses in this preclinical model, and that the observed responses may vary across the tested formulation conditions.

The in vitro results indicate that *L. paracasei* Yb interacts with both innate and lymphoid immune cells. RAW 264.7 macrophages exhibited increased levels of TNF-α across all tested concentrations, elevated IL-1β at the highest concentration, and increased IL-10 at the two higher concentrations. Because macrophages can contribute to both inflammatory activation and immune regulation, the upregulation of TNF-α or IL-1β should not be interpreted solely as a beneficial anti-allergic effect; rather, these responses indicate immune cell activation and recognition of bacterial stimuli [23]. The splenocyte assay further showed increased TNF-α, IFN-γ, and IL-10 compared to the untreated control, although the responses did not follow a straightforward dose-dependent pattern. This pattern is consistent with previous studies showing that lactobacilli can modulate cytokine production in murine immune cells, including IL-10 and IFN-γ responses in vitro [24]. In addition, studies on other *L. paracasei* strains have shown that this species can induce cytokine- and chemokine-mediated immunomodulatory responses in both in vitro and in vivo models [6]. Taken together, the present in vitro findings support the immunological responsiveness of macrophages and splenocytes to *L. paracasei* Yb and are consistent with the broader concept that lactobacilli can regulate cytokine secretion by immune cells. However, these in vitro cytokine responses should be interpreted as supportive cellular evidence and not as direct proof of the mechanisms underlying the in vivo outcomes observed in the OVA-induced allergic airway inflammation model.

In the OVA-induced model, serum total IgE and OVA-specific IgE levels confirmed systemic immunoglobulin responses after sensitization and challenge. The Negative control group and YG-FD group showed higher total IgE than the Control group, and all OVA-sensitized groups showed increased OVA-specific IgE compared with the Control group. YG and BP had intermediate total IgE levels; their OVA-specific IgE levels were lower than those of YG-FD. However, neither YG nor BP differed significantly from the Negative control group in total IgE or OVA-specific IgE. These findings suggest that *L. paracasei* Yb did not markedly suppress serum IgE responses under the current experimental conditions. Previous research indicates that selected probiotic strains can reduce IgE-related responses and allergic airway inflammation in murine models, including *L. paracasei* L9 and *Lactobacillus paragasseri* BBM171 [25,26]. The relatively modest IgE response observed in this study may be attributable to strain-specific effects, variations in dosage and delivery format, the timing of administration, differences in the OVA model, or the limited sample size.

The histological findings provided the clearest tissue-level evidence of formulation-associated responses. The Control group showed no remarkable histopathological findings, whereas the Negative control group exhibited marked lung inflammation characterized by inflammatory cell infiltration and granulomatous inflammatory responses. YG and BP significantly reduced the lung inflammation score compared with the Negative control group, while YG-FD showed an intermediate score. Because the histological score integrates inflammatory cell infiltration and granulomatous inflammatory responses, it may capture cumulative tissue injury more directly than a single serum or BALF endpoint. These results suggest that YG and BP were associated with attenuation of lung inflammatory lesions in this OVA-induced allergic airway inflammation model. Nevertheless, because the improvement was not equally reflected in airway responsiveness or BALF eosinophil proportions, the histological result should be interpreted as tissue-level evidence rather than as proof of broad functional recovery.

The splenocyte cytokine results were consistent with selective modulation of Th2-associated immune responsiveness. Under unstimulated conditions, IL-4, TNF-α, and IL-6 did not differ significantly among groups. After ConA stimulation, the Negative control group had the highest IL-4 level, whereas YG and BP significantly reduced IL-4 secretion. Because IL-4 is a central Th2 cytokine involved in allergic sensitization and IgE class switching, lower ConA-induced IL-4 production in YG and BP groups is consistent with a reduced Th2-prone response [4,26]. In contrast, TNF-α and IL-6 did not differ significantly among groups after ConA stimulation. This is important because it prevents overinterpreting the probiotic effect as a broad suppression of inflammatory cytokines. Instead, the data suggest a more selective modulation of IL-4-associated splenocyte responsiveness. The absence of parallel significant reductions in serum IgE also indicates that ex vivo cytokine modulation and systemic antibody responses may not fully overlap within the time frame and sensitivity of the present model.

In this study, formulation refers to the administered product form of *L. paracasei* Yb rather than the yogurt matrix alone. The formulation-related findings should be interpreted in the context of practical probiotic product development. Previous studies have shown that food matrices and processing conditions can influence probiotic viability, stress tolerance, metabolic activity, and host interaction [12,15]. Formulation- or product-dependent probiotic effects have also been reported in other experimental models. For example, *Lactobacillus casei* BL23 showed a stronger protective effect when delivered in milk than in a nutrient-free buffer in a colitis model [13], and the beneficial properties of a BB-12 and LA-5 probiotic mixture varied according to the administration matrix [14]. Studies on yogurt or fermented dairy products further support the view that product composition and processing conditions influence probiotic viability and functional outcomes [27,28]. Moreover, Al-Sahlany et al. reported that incorporation of date juice into a bio-yogurt formulation altered its physicochemical properties, improved probiotic viability during refrigerated storage, and affected hematological and serum biochemical parameters in mice [29]. In addition, evidence from an LPS-induced rat model showed that purified bacterial-derived exopolysaccharides and yogurt fermented with the EPS-producing strain did not produce equivalent cytokine profiles [30]. Together, these findings illustrate that fermented dairy formulations should be considered complete product systems rather than simple carriers of bacterial cells or isolated bacterial-derived components. In the present study, differences among the tested formulations were most evident in lung histology and ConA-induced IL-4 secretion, whereas other endpoints showed only numerical or non-significant changes. Thus, the results are best described as formulation-associated responses under the administered dosing conditions, rather than as evidence that the yogurt matrix alone caused the observed effects or that any single formulation was superior across all endpoints.

Several structural features of our experimental design provide critical context when interpreting these results. First, this study did not include a probiotic-free yogurt placebo control; hence, the effects observed in the YG group cannot be attributed solely to *L. paracasei* Yb, the yogurt matrix, or their interaction. Instead, they should be interpreted as the overall response associated with the tested yogurt-based formulation. Second, the administered CFU levels were not fully equivalent among the YG, YG-FD, and BP groups due to the physical delivery constraints outlined in Section 2.2. Intriguingly, however, our data suggest that a simple linear CFU dose–response model does not adequately explain the outcomes. The BP group delivered the highest viable cell dose, whereas the YG group had the lowest CFU, yet both formulations achieved statistically significant reductions in lung histological inflammation scores and suppressed Th2-associated ex vivo IL-4 secretion. Conversely, the YG-FD formulation, despite supplying an intermediate viable count, did not reach statistical significance in these endpoints. These observations suggest that formulation-specific factors beyond CFU dose, such as bacterial physiological state, processing-related stress, post-ingestion reactivation, and gastrointestinal exposure, may have contributed to the observed differences among groups. The weaker and less consistent response observed in the YG-FD group cannot be attributed merely to cell count, suggesting that downstream processing and formulation conditions may have contributed to functional differences. While we did not evaluate gastrointestinal survival, fecal retrieval, or microbiome profiles—preventing us from isolating their specific contributions—these findings nonetheless provide preclinical evidence that formulation and processing conditions are key factors shaping the functional performance of *L. paracasei* Yb. In addition, the relatively small sample size may have reduced the statistical power to detect differences in some in vivo immunological outcomes, particularly those showing numerical but non-significant trends. Finally, all in vivo data were obtained from a mouse model, and no human data are available for *L. paracasei* Yb in asthma or allergic airway inflammation. Therefore, the clinical relevance of these findings requires further validation in appropriately powered preclinical studies and controlled human studies.

A strength of this study is that it evaluated the same probiotic strain in three administration formats relevant to practical product development: fresh yogurt, freeze-dried yogurt, and bacterial powder. This design allowed the responses associated with these practical *L. paracasei* Yb formulations to be assessed within the same experimental framework. The study also integrated in vitro immune cell assays with multiple in vivo endpoints, including serum immunoglobulins, airway responsiveness, lung histology, splenocyte cytokines, and BALF immune cells. This broader assessment reduces reliance on a single outcome and provides a more balanced view of formulation-associated responses. Because the test formulations differed in processing methods and viable cell counts, these results are best interpreted as a practical performance evaluation under the tested dosing conditions. Consequently, isolating matrix-specific effects, achieving strict dose equivalence, and elucidating underlying mechanisms remain important objectives for future research.

## 5. Conclusions

This study showed that live *Lacticaseibacillus paracasei* Yb can activate immune cell responses in vitro and that practical *L. paracasei* Yb formulations were associated with distinct response patterns in an OVA-induced allergic airway inflammation mouse model. The in vivo findings were not uniformly significant across all endpoints, but differences were observed in lung histological scores and ConA-induced IL-4 production, whereas serum IgE, OVA-specific IgE, airway responsiveness, and BALF eosinophils showed modest or non-significant changes. These results support the preclinical immunomodulatory potential of *L. paracasei* Yb and suggest that practical formulation conditions may shape the observed response patterns under the tested dosing conditions. In summary, rather than providing evidence of a matrix-specific effect alone, our findings offer a practical comparison of formulations under feasible dosing conditions, in which processing pathways and viable counts varied. Further CFU-matched studies incorporating probiotic-free yogurt controls, gastrointestinal survival and fecal recovery assays, microbiome profiling, and controlled clinical validation are needed before clinical relevance or application can be inferred.

## Figures and Tables

**Table 1:** Daily administration doses and viable counts of *Lacticaseibacillus paracasei* Yb formulations used for mouse feeding.

Group	Administration Material	Administered Amount per Mouse	Dose Normalized to Body Weight	Administered viable Count per Mouse *	Viable Count Normalized to Body Weight
Control	Sterile water	0.4 mL/day	20 mL/kg	N/A	N/A
Negative control	Sterile water	0.4 mL/day	20 mL/kg	N/A	N/A
YG-FD	Freeze-dried *L. paracasei* Yb yogurt	164 mg/day	8200 mg/kg	4.8 × 10^7^ CFU/day	2.4 × 10^9^ CFU/kg
YG	*L. paracasei* Yb yogurt	0.4 mL/day	20 mL/kg	2.4 × 10^7^ CFU/day	1.2 × 10^9^ CFU/kg
BP	*L. paracasei* Yb bacterial powder	0.94 mg/day	47 mg/kg	7.6 × 10^8^ CFU/day	3.8 × 10^10^ CFU/kg

* The daily administered amounts and viable counts were determined based on the feasible administration volume and formulation-specific preparation conditions for each commercially feasible formulation type. For the YG-FD group, the administered amount was constrained by the maximum feasible reconstitution concentration of the freeze-dried yogurt and the allowable oral gavage volume for mice. The YG group was administered in its original yogurt form within the allowable daily gavage volume, whereas the BP group was prepared as a concentrated bacterial powder suspension and therefore delivered the highest viable count among the tested products. Consequently, the dosing conditions in this study reflect practical, formulation-specific constraints rather than a CFU-matched dosing design. CFU, colony-forming units.

## Data Availability

The original contributions presented in this study are included in the article. Further inquiries can be directed to the corresponding author.
